# Trochanteric fracture following hip arthrodesis: case presentation

**DOI:** 10.1002/ccr3.1275

**Published:** 2017-12-07

**Authors:** Bogdan Deleanu, Radu Prejbeanu, Dinu Vermesan, Horia Haragus, Lucian Honcea, Mihail‐Lazar Mioc, Eleftherios Tsiridis, Vlad Predescu

**Affiliations:** ^1^ 1st Orthopedics and Traumatology Clinic Emergency Clinical County Hospital Timisoara Timisoara Romania; ^2^ “Victor Babes” University of Medicine and Pharmacy Timisoara Timisoara Romania; ^3^ Aristotle University of Thessaloniki Thessaloniki Greece; ^4^ St. Pantelimon Emergency Hospital “Carol Davila” University of Medicine and Pharmacy Bucharest Romania

**Keywords:** Ankylosis, arthrodesis, plate, trochanteric

## Abstract

Even if the intertrochanteric fracture under an arthrodesis hip is rare and the optimal surgical treatment is controversial, we consider that treating this kind of fracture with a locked plate was a success.

## Introduction

Lately, an increase in incidence regarding hip fractures in developed countries has been noticed. Despite this, hip fractures following coxofemoral arthrodesis are rare cases needing special attention. This is strongly related with the fact that conventional treatment methods are not usually applicable.

In our manuscript, we shall present a patient with an intertrochanteric fracture which occurred on a hip that had a previous arthrodesis surgery.

Few cases of proximal femur fractures in patients with hip ankyloses were reported in the last year. The treatment options for those cases included using either plates or intramedullary nails for the osteosynthesis 1, 2.

## Case Presentation

**Figure 1:**
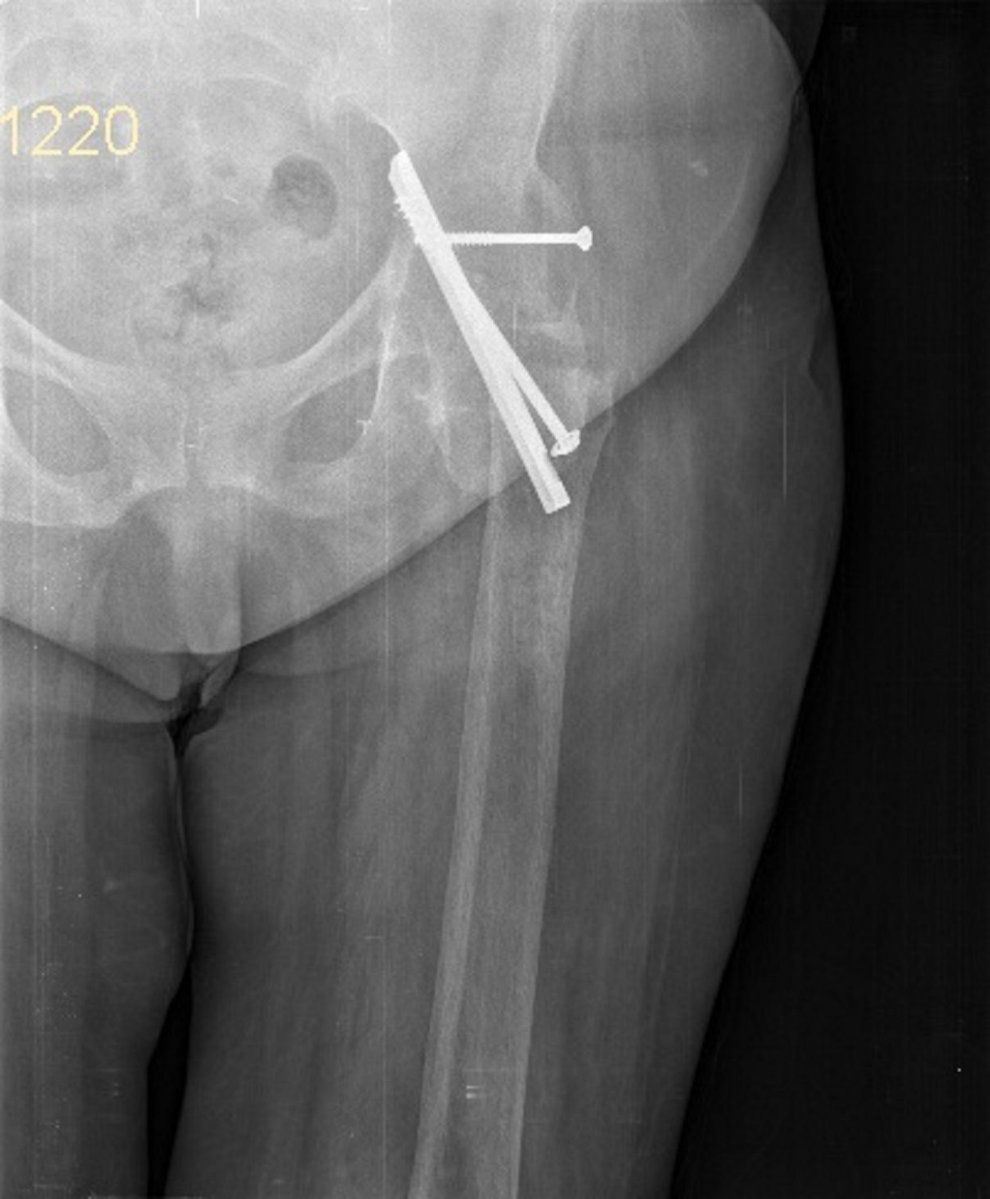
Preoperative X‐ray.

**Figure 2:**
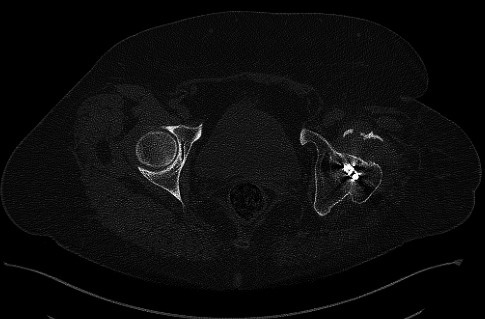
Preoperative CT‐scan.

**Figure 3:**
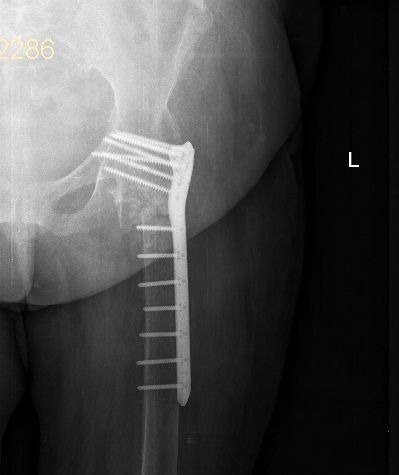
Postoperative X‐ray.

A 62‐year‐old woman was referred to the emergency department in our hospital due to a body height fall. She was presented with severe pain in her left hip and the inability to walk. Her following examination revealed a 30‐year‐old femoral neck fracture. The option of treatment at that time was an arthrodesis of the left hip. Having presented with the history of trauma, we did not take into consideration a proximal femoral malignancy. However, Ungureanu et al. stated in their study that tumoral growths can be kept hidden under adipose tissue or local swelling located at the thigh level and they could appear concurrently with trauma 3. Therefore, the differential diagnosis was made using the patient history and the CT description given by the radiology specialist. A standard emergency anteroposterior radiograph revealed an intertrochanteric fracture, severe deformity of the left hip joint, and the migrated implants used for the arthrodesis (Fig. 1). Computed tomography (CT) imaging of the left hip joint showed a displaced intertrochanteric fracture distal to the ankylosed hip joint, some artifacts due to the osteosynthesis material, and marked atrophy of the gluteus muscles (Fig. 2). Due to the rarity of the pathology, several discussions with fellow surgeons were needed to establish what the best therapeutic option was. We decided to perform a surgery to remove the osteosynthesis material used for arthrodesis of the left hip and then perform the internal fixation using a locking plate and locked screws (Fig. 3). The patient was positioned in dorsal decubitus; we used a lateral approach (a skin incision reaching approximately 5 cm proximal and 15 cm distal from what used to be the tip of the greater trochanter). Intraoperative passive mobilization of the limb was realized to outline some intraoperative anatomical landmarks. After initial hardware removal, normal femoral rotation was re‐established and checked intraoperatively using the C‐arm. A rigid secondary fixation was realized using a locked plate and 13 locking screws. Postoperatively, the patient's evolution was good, and after 2 weeks, she could walk using double crutches. Postoperatively after 3 months, the fracture showed signs of consolidation.

## Discussion

We all agree that treatment in a 62‐year‐old patient with an intertrochanteric fracture and no other associated diseases should be surgical and performed as soon as possible. There are several options for surgical treatment of this kind of fracture including the dynamic hip screw, a short intramedullary nail, cannulated screws, an AO proximal femoral plate, or sliding plates.

The particularity of this case is represented by the pre‐existed coxofemoral ankyloses. This type of fracture is rare, the experience of orthopedic surgeons is low, and the literature is insufficient regarding this topic.

The problem with deciding the correct surgical treatment and implant is caused by the small number of similar cases and thus resulting in a lack of literature and experience.

For this patient, we decided to use a locking plate because the changes produced on a 30‐year‐old hip arthrodesis influence the choice of surgical treatment of such a fracture. These changes included the atrophy of muscles around the hip and an abnormal lower limb biomechanics, together with femoral head ankylosis. The reasoning behind our choice was that we could not approach the hip from the usual intramedullary nail insertion point due to joint rigidity (caused by ankylosis and muscle atrophy and retraction). Also, we wanted to avoid the blade plate due to the big bone stock it takes out of the femoral neck and head.

Manzotti et al. presented a case report with an intertrochanteric fracture of an arthrodesed hip treated successfully using a double‐plating technique with 4.5‐mm titanium reconstruction plates 1. Another case presented by Darwish et al. introduced the use of a single heavy duty locked plate and cannulated screws to treat the fracture 4.

Compression screws do not offer rigid fixation and the possibility for early weight bearing; therefore, we did not consider that as a good surgical choice for this kind of fracture in our case 5, 6.

We discussed the option of performing total hip arthroplasty for our patient. The reason for not opting for this type of treatment was the high failure rates of hip arthroplasty in patients with muscle atrophy, ranging from 14% to 29%, as presented in several studies 7, 8.

Some authors consider that the best choice of treatment in proximal femoral fracture on an arthrodesed or ankylosed hip is an intramedullary nail along with a gamma nail 5, 9, 10. The abduction stiffness of our patient's hip prevented us from obtaining a good position for implant insertion through the tip of the trochanteric area. This often happens in normal intertrochanteric fractures on patients with advanced hip arthrosis. In response to this complication, surgeons often chose to use extramedullary implants such as sliding plates or blade plates.

## Conclusion

Even if the intertrochanteric fracture under an arthrodesis hip is rare and the optimal surgical treatment is controversial, we consider that treating this kind of fracture with a locked plate was a success. The biomechanical properties of the implant we used, allow it to act both as an internal fixator for the trochanteric fracture and as a rigid implant for re‐establishing the arthrodesis with long locked screws.

Such difficult cases to treat often require working as a team together with experienced radiologists and other fellow surgeons, to establish the best therapeutic option. Even if the treatment plan can sometimes feel very complicated, the development of newer implants has allowed for elaborate surgical interventions.

It is hard to say that a surgeon can develop experience when it comes to specific cases like this one, but more than everything, that surgeon needs to properly prepare that patient for the upcoming treatment. This includes quality imaging, extensive investigation on specific literature, asking advice from fellow surgeons, and planning the surgery.

Future cases must be described in the literature to offer as many surgical options as possible as implants continue to advance. This leaves the place opened for a case series article, with longer follow‐up periods.

## Authorship

BD, RP, DV, HH, LH, M‐LM, ET, and VP: analyzed the data. BD, RP, HH, LH, and M‐LM: collected the data. BD: involved in study conception. LH and M‐LM: wrote and verified the manuscript, respectively.

## Conflict of Interest

None declared.
